# Constitutive Serotonin Tone as a Modulator of Brown Adipose Tissue Thermogenesis: A Rat Study

**DOI:** 10.3390/life13071436

**Published:** 2023-06-25

**Authors:** Maja Kesić, Petra Baković, Vladimir Farkaš, Robert Bagarić, Darko Kolarić, Jasminka Štefulj, Lipa Čičin-Šain

**Affiliations:** 1 Department of Molecular Biology, Ruđer Bošković Institute, HR-10000 Zagreb, Croatia; maja.kesic@irb.hr (M.K.); petra.bakovic@mef.hr (P.B.); 2 Department of Experimental Physics, Ruđer Bošković Institute, HR-10000 Zagreb, Croatia; vfarkas@vef.hr (V.F.); robert.bagaric@irb.hr (R.B.); 3 Centre for Informatics and Computing, Ruđer Bošković Institute, HR-10000 Zagreb, Croatia; dkolaric@irb.hr

**Keywords:** serotonin, brown adipose tissue, adaptive thermogenesis, obesity, body weight homeostasis, energy balance, rat model, cold exposure, β-adrenergic agonist

## Abstract

Brown adipose tissue (BAT), an important regulator of thermogenic and metabolic processes, is considered a promising target to combat metabolic disorders. The neurotransmitter and hormone serotonin (5HT) is a major modulator of energy homeostasis, with its central and peripheral pools acting in opposing ways. To better understand how individual variations in 5HT homeostasis influence the thermogenic functionality of BAT, we used a rat model consisting of two sublines with constitutively increased (high-5HT) or decreased (low-5HT) whole-body 5HT tone, developed by selective breeding for platelet 5HT parameters. We have shown that animals with constitutively low 5HT activity maintained at a standard housing temperature (22 °C) have greater interscapular BAT (iBAT) mass and higher iBAT metabolic activity (as evidenced by measurements of iBAT temperature and glucose uptake), accompanied by increased iBAT mRNA expression of key thermogenic genes, compared to animals with high 5HT tone. In response to further thermogenic challenges—intermittent cold exposure or treatment with a β_3_-adrenergic agonist—5HT sublines show several functional and molecular differences linking constitutively low endogenous 5HT tone to higher BAT activity/capacity. Overall, the results support a role of 5-HT in the control of BAT thermogenesis They also suggest that individuals with lower 5HT activity may be more sensitive to β3-adrenergic drugs.

## 1. Introduction

According to the 2022 World Health Organization report, more than 1 billion people worldwide are obese [1]. Living with obesity poses a huge risk of a range of physical and mental health problems.

Obesity is generally the result of a chronic imbalance between energy intake and energy expenditure [2]. Lifestyle factors such as the excessive consumption of high-calorie foods and a sedentary lifestyle are well-established contributors to energy imbalance. In addition, various biological factors such as genetics and the constitutive activity of systems that regulate appetite and/or metabolism can also contribute to energy imbalance. Therefore, to prevent or manage obesity, it is important to address both lifestyle factors and the underlying biological mechanisms. Pharmacotherapeutic interventions aimed at biological systems that regulate food intake, despite considerable efforts, have not produced satisfactory results [3], and now much hope is placed on therapies targeting biological mechanisms that regulate energy expenditure, as an alternative strategy for preventing weight gain and/or inducing weight loss [2,4,5].

Total body energy expenditure comprises three major components: basal metabolic rate, physical activity and (adaptive) thermogenesis. Adaptive thermogenesis refers to the production of heat in response to external stimuli such as nutrition or exposure to temperature below thermoneutrality. This process can account for up to 20% of total daily energy expenditure [6] and occurs mainly in specialized brown adipose tissue (BAT). For many years, it was believed that BAT in humans occurs only in newborns, where it is important for maintaining core body temperature [7]. However, the discovery of the active BAT depot in adults [8,9] and further clinical evidence of inverse correlation of its mass/activity with body mass index and fat mass [10,11], blood glucose levels [11], and diabetes status [12] suggest that increasing energy expenditure through activation of BAT thermogenesis could help protect against obesity and related metabolic diseases [13,14,15].

In contrast to white adipocytes, in which excess chemical energy is stored as triglycerides, brown adipocytes utilize chemical energy from fatty acids and glucose to produce heat [13,16]. This process relies on the activity of the BAT-specific mitochondrial uncoupling protein-1 (UCP1), which is localized in the inner mitochondrial membrane and converts chemical energy into heat. Various thermogenic stimuli that are sensed by the brain hypothalamic nuclei activate the sympathetic nervous system [7,17] and increase the release of noradrenaline within BAT, which is densely innervated by adrenergic fibers [18]. By acting on brown adipocytes through β_3_-adrenergic receptors, noradrenaline stimulates the mitochondriogenesis and synthesis of UCP1 [19]. In addition to neuronal mechanisms, BAT functions are regulated by metabolic hormones and various paracrine/autocrine factors [13,17,20,21,22].

Serotonin (5-hydroxytryptamine, 5HT) is a signaling molecule important in the regulation of energy homeostasis by controlling many behavioral and physiological processes [23]. Serotonergic regulation takes place through two functionally independent 5HT compartments—central and peripheral—in which 5HT actions lead to the opposite effects on systemic energy balance. Centrally acting 5HT, synthesized in the brainstem, modulates brain functions to inhibit food intake and reduce body weight [24,25], while peripheral 5HT, derived mainly from the gut, acts to increase adiposity and body weight [26,27] by regulating lipogenesis and lipolysis, insulin secretion, gluconeogenesis, and thermogenesis (reviewed in [27,28,29]).

Recent studies have shown that manipulating 5HT activity can have significant effects on BAT mass and function. Increasing 5HT activity by pharmacological blockade or genetic deletion of the 5HT transporter (5HTT), which is the main regulator of extracellular 5HT bioavailability, has been shown to reduce BAT mass and impair BAT function [30]. It was also shown that 5HT inhibits brown adipocyte differentiation in vitro [31] and β_3_-adrenergic induced thermogenic activation in vivo [32]. In contrast, pharmacological inhibition of tryptophan-hydroxylase 1 (Tph1), a rate-limiting enzyme in peripheral 5HT synthesis, increased BAT mass and enhanced BAT adaptive thermogenesis [32,33]. Interestingly, cell-type specific deletion of *Tph1* in white/brown adipocytes protects mice from high-fat diet-induced obesity by increasing their energy expenditure [32,33]. However, the gut-specific deletion of *Tph1* has no effect on systemic energy metabolism in this context [34]. Furthermore, in contrast to peripheral 5HT which inhibits BAT thermogenic activity, the centrally acting amine can promote BAT-adaptive thermogenesis [35]. These findings underscore the complexity of 5HT regulation of BAT functions at the level of whole organism, highlighting the need for further studies to better understand the role of 5HT in this context. Although the number of papers investigating BAT functioning has grown enormously in recent years, those related to 5HT are scarce. In our studies on serotonergic regulation of energy homeostasis, we use a genetic model of rats with constitutively altered 5HT homeostasis (Wistar–Zagreb 5HT, WZ-5HT rats) [36,37,38]. The model consists of two rat sublines—high-5HT subline (H) and low-5HT subline (L)—with approximately double, lifelong differences in circulatory 5HT levels. The animals of the two sublines also exhibit constitutive differences in the activity of both peripheral and central 5HT systems, as indicated by neuro/biochemical, pharmacological, behavioral, and other functional studies [38,39,40,41,42,43]. Therefore, within the context of integrative physiology, animals from the high-5HT subline are considered constitutively hyperserotonergic compared to animals from the low-5HT subline.

**Figure 1:**
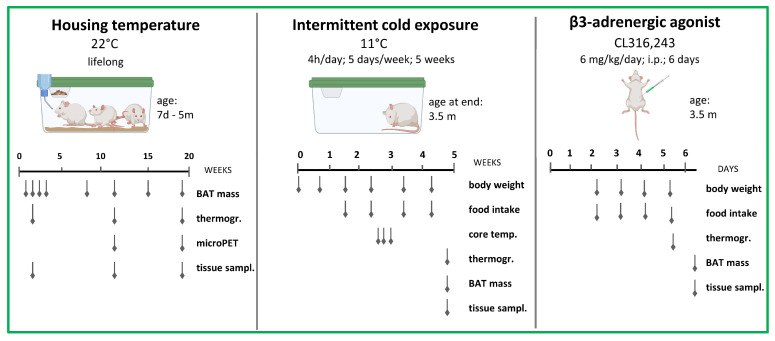
A schematic diagram showing the main experiments and indicating the timing of the different measurements performed on the 5HT sublines. Created by BioRender.

In our previous research, we have used the WZ-5HT rat model to investigate the impact of interindividual differences in 5HT tone on the net energy balance of the organism. We have shown that constitutively higher 5HT activity is associated with increased body adiposity and impaired glucose homeostasis [44], but, interestingly, with less adverse metabolic outcomes in response to a high-fat diet [45]. Baseline measurements have also shown that high-5HT animals have a lower amount of BAT [46] and a lower skin temperature above BAT [45], suggesting that differences in thermogenesis contribute to differential metabolic health observed between the 5HT sublines. In the present study, we aimed to further investigate the interplay between endogenous 5HT tone and BAT functioning by comparatively analyzing the BAT thermogenic activity (at functional and molecular levels) of 5HT sublines in response to specific physiological (cold exposure) or pharmacological (β_3_-adrenergic agonist) challenges (Figure 1).

## 2. Materials and Methods

### 2.1. Animals

Studies were performed on two sublines of Wistar–Zagreb 5HT (WZ-5HT) rats developed by selective breeding for the extreme values of platelet serotonin level (PSL) and velocity of platelet serotonin uptake (PSU). The development of a high-5HT subline and a low-5HT subline of WZ-5HT rats has been described previously [36,37,38,47].

Male animals aged 7 days to 5 months (as indicated in the figure legends) were used in the present study. Unless otherwise indicated, animals were housed three per cage under controlled conditions (temperature, 22 ± 2 °C; humidity, 55 ± 10%; 12-h light–dark cycle) with food and water ad libitum. In experiments with prepubertal animals (age 7 to 30 days) we ensured that the day and time of birth were identical for animals from 5HT sublines. In addition, within 24 h from birth litters from high-5HT and low-5HT, the mothers were culled (balancing the number of males and females) to ensure that the 5HT sublines had equal numbers of pups/litter. In all experimental procedures, high-5HT and low-5HT animals were always alternated.

### 2.2. Cold Exposure Experiments

Cold exposure experiment in the neonatal period was performed on three litters per subline, with an equal number of pups/litter in each subline. In brief, pups at 7 days of age were placed in a cage without bedding at an ambient temperature of 17 °C and kept there for one hour before their skin temperature over the interscapular BAT (iBAT) was measured using infrared thermography (see Section 2.4).

In the experiment measuring iBAT glucose uptake after the first and second exposure to cold, adult (5-month old) animals acclimated to room temperature (RT) were placed individually in cages without bedding and kept for 24 h at a low ambient temperature (12 °C), upon which the glucose uptake was determined using ^18^FDG-microPET imaging (see Section 2.5). Animals were then housed at RT for the next 7 days, following which the cold exposure and glucose uptake measurement were repeated in the same way as in the first exposure.

In the chronic cold exposure (CE) experiment, adult (2.5-month old) animals from the high-5HT and low-5HT sublines were randomly divided into control groups (H-RT, L-RT) and cold exposure groups (H-CE, L-CE). For the next 5 weeks, animals from the H-RT and L-RT groups were kept all the time at room temperature, while animals from the H-CE and L-CE groups were subjected to intermittent exposure to low ambient temperature. The procedure for intermittent cold exposure was standardized as follows: 5 days per week (Monday to Friday), animals were transferred to cages without bedding and placed for 4 h (typically from 9 am to 1 pm) in a cold chamber with a temperature of 11 °C, without food and water. After that, the animals were returned to their home cages and to standard housing temperature. Control animals from both 5HT sublines were placed in a clean cage and kept in an adjacent room with a temperature of 22 °C for 4 h to ensure that they were exposed to similar disturbances (noise, handling).

The body weight gain and food intake of all animals were monitored once per week. At 9 am, rats were presented with a preweighed amount of food. After 24 h, the remaining food was weighed, and the food consumed was obtained by subtracting the uneaten food. Core body temperature was measured with a rectal rat probe (Physitemp Instruments, Clifton, NJ, USA) on three consecutive days during the third week of the experiment, immediately after cold exposure. Shivering was visually checked by the experimenter. On day 31 of the experiment (after 24 CE episodes), the temperature of the skin over the interscapular region, indicating iBAT thermogenic activity, was measured immediately after cold exposure using infrared thermography, as described below (see Section 2.4). After five weeks (25 episodes) of intermittent cold exposure, animals were euthanized and their total iBAT and gonadal white adipose tissue (gWAT) was manually dissected and wet-weighed. Small portions of iBAT were excised (see Section 2.6) and stored for further analyses. Control animals of both sublines were subjected to exactly the same procedure, except for cold exposure.

### 2.3. Pharmacological Experiments

In the experiment measuring iBAT glucose uptake after a single administration of selective β_3_-adrenergic agonists, 3-month-old males acclimated to RT were injected intraperitoneally BRL 37344 (Sigma-Aldrich, St. Louis, MO, USA) at a dose of 1 mg/kg body weight; control animals received saline. Thirty minutes after the injection, iBAT glucose uptake was determined by FDG-microPET imaging as described below (see Section 2.5).

In the experiment investigating functional and molecular response to the repeated administration of selective β_3_-adrenoceptor agonists, 3.5-month-old animals from each subline were randomly assigned to treatment groups (H-CL, L-CL), receiving an intraperitoneal injection of CL316,243 (Tocris Biosciences, Bristol, UK) at a dose of 6.0 mg/kg body weight for 6 consecutive days, with the control groups (H-sal, L-sal) receiving the same volume of physiological saline. The body weight gain and food intake of all animals were measured after the 2nd, 3rd, 4th, and 5th injection. Two hours after the 5th injection, the iBAT temperature was measured using infrared thermography as described (see Section 2.4). A full 24 h after the last (6th) injection, animals were euthanized and the BAT tissue was collected as described below.

### 2.4. Infrared Thermography

Thermogenic activity of the iBAT was assessed by infrared thermography. The day before thermographic imaging, animals (adults) were anesthetized (SomnoSuite, Kent Scientific, Torrington, CT, USA) with isoflurane and the fur over the iBAT area was shaved to expose the skin for temperature readings in the iBAT. The next day, the lightly anesthetized animals were elastically fixed on a marble base to ensure a similar position of all animals during the thermographic recording by the FLIR T335 digital infrared camera (FLIR Systems Inc, Wilsonville, OR, USA). Three to four thermographic images of each animal were taken and analyzed using ThermoMED software, which we had developed previously [48]. 

### 2.5. ^18^F-FDG-microPET Imaging

Positron emission tomography (PET) scans were obtained with the ClearPET, a high-performance small animal PET scanner (Elysia-raytest GmbH, Straubenhardt, Germany). Animals were anesthetized under 4% isoflurane in 100% oxygen, weighed and injected with 40 MBq of [^18^F]fludeoxyglucose (FDG) via the tail vein. After 30-min uptake time, the rats were anesthetized again and placed into the PET scanner with the iBAT in the center of the field of view and scanned during the 40-min (20 two-minute frames). Animal temperature and breathing were monitored throughout the procedure. Post-imaging reconstruction was conducted using OSMAPOSL iterative reconstruction algorithm incorporated in commercial ClearPET image reconstruction software. In order to obtain and compare the data from iBAT, the equal size volumes of interest (VOI) were manually drawn around the visible iBAT of each animal. The ^18^F-FDG uptake of iBAT was analyzed using PMOD 3.2 image analysis software (PMOD Technologies LLC, Zürich, Switzerland) and quantified as standard uptake value (SUV). SUV was calculated by the formula (measured activity, MBq/mL)/[(total activity, MBq)/(body weight, g)]. 

### 2.6. Tissue Collection for Molecular Analyses

Rats were euthanized by an inhalant anesthetic (soflurane) overdose (SomnoSuite, Kent Scientific, Torrington, CT, USA), and iBAT (cca 30 mg/sample) and gWAT (cca 100 mg/sample) samples were collected, weighed, and stored in RNAlater solution (Qiagen, Venlo, The Netherlands) for subsequent mRNA expression analyses. In addition, samples of iBAT (200–300 mg) were collected for the subsequent determination of the protein levels of UCP1, the major marker of adaptive thermogenesis, and the levels of noradrenaline, the marker of adrenergic activation, and immediately frozen on dry ice for the subsequent determination of protein levels. All tissue samples were stored at −80 °C until further analysis.

### 2.7. mRNA Expression Studies

Total RNA was isolated from iBAT samples using the RNeasy Mini Kit (Qiagen, Germantown, MD, USA) according to the manufacturer’s protocol including on-column DNA digestion step. The homogenization of the tissue samples and RNA quality control were performed as earlier described [44]. The relative levels of specific mRNAs were determined by reverse transcription-quantitative real-time PCR (RT-qPCR) using SYBR Green detection chemistry, as described in our previous study [44], with actin beta (Actb) used as a reference gene. The sequences of the primers used in qPCR are listed in Table S1.

### 2.8. ELISA Measurements

BAT samples for UCP1 and noradrenaline measurements were homogenized in tissue protein extraction reagent (T-PER, Thermo Scientific, Waltham, MA, USA) supplemented with protease inhibitor (Halt Protease Inhibitor Cocktail, Thermo Scientific, Waltham, MA, USA). After centrifugation (10,000× *g*, 20 min, +4 °C), the supernatants were used to determine the levels of UCP1 and noradrenalin using enzyme-linked immunosorbent assays (ELISA), according to the manufacturers’ protocol (ElabScience, Wuhan, China and Demeditec Diagnostics, Kiel, Germany, respectively). Levels of UCP1 and noradrenaline were expressed per total protein content determined by the Bradford method.

### 2.9. Statistical Analysis

GraphPad Prism v8.4.3 (GraphPad Software, San Diego CA, USA) was used for statistical analyses and the visualization of the results. Normality of data was tested by D’Agostino–Pearson omnibus test and the homogeneity of variances by Bartlett’s test. Outliers, detected using the Grubbs method, were excluded from the analysis. Normally distributed data were analyzed using two-tailed unpaired Student’s *t*-test (with Welch’s correction if variances differed significantly), or one-way analysis of variance (1w-ANOVA) with Fisher’s least significant difference (LSD) post-hoc test. Non-normally distributed data were analyzed using the Mann–Whitney (MW) test or Kruskal–Wallis (KW) with Dunn’s post-hoc test. A paired *t*-test was used to compare animals after first and second cold exposure. Total area under curve (AUC) was calculated using the linear trapezoidal method.

Results are presented as individual values and/or group means with standard deviation (SD) or standard error of the mean (SEM). Differences were considered statistically significant when *p* < 0.05. *p*-values < 0.1 were considered a trend and are shown in brackets.

## 3. Results

### 3.1. Basal Characteristics of the Study Animals

**Figure 2:**
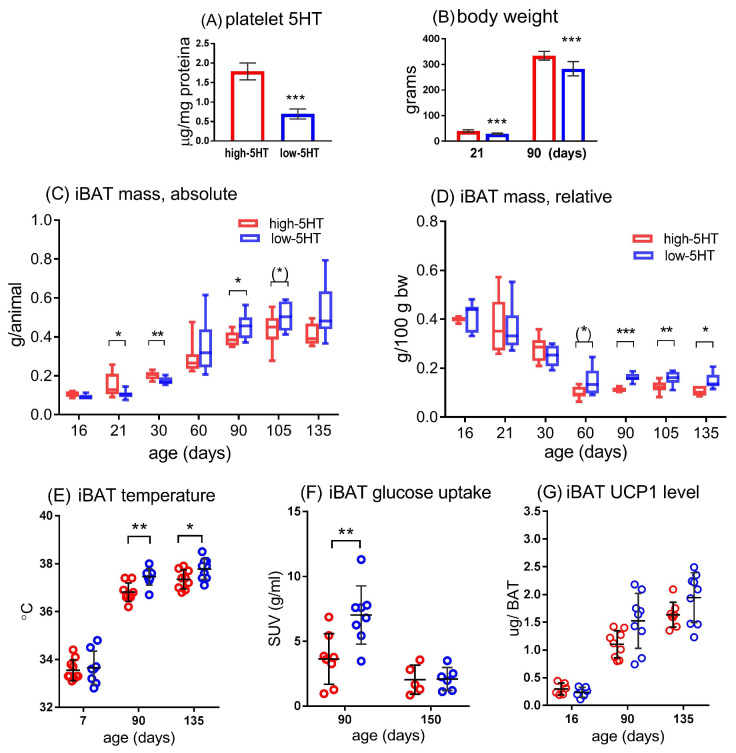
Basal characteristics of high-5HT and low-5HT animals housed at an ambient temperature of 22 °C. (**A**) Platelet serotonin (5HT) levels in high-5HT and low-5HT animals, typical values; *n* = 12/group; (**B**) Body weight of animals from 5HT sublines at 21 and 90 days of age, typical values; *n* = 12/group; (**C**,**D**) Mass of interscapular brown adipose tissue (iBAT) in high-5HT and low-5HT animals at different ages, expressed as weight per animal (**C**) or weight per body mass (**D**), *n* = 6 (16-day-old) or 8–9/group; (**E**–**G**) Comparison of iBAT characteristics between high-5HT (red circles) and low-5HT (blue circles) animals of different age in terms of skin temperature above iBAT measured by infrared thermography (**E**), iBAT glucose uptake measured by FDG-microPET imaging (**F**) and uncoupling protein 1 (UCP1) concentration measured by ELISA (**G**); *n* = 5–10/group. Mean ± SD (**A**,**B**,**E**–**G**) or median, interquartile range and min to max (**C**,**D**) are presented; * *p* < 0.05, ** *p* < 0.01, *** *p* < 0.001; *p*-values obtained by *t*-test or MW test, as appropriate; iBAT = interscapular brown adipose tissue, SUV = standard uptake value, UCP1 = uncoupling protein 1.

Consistent with our previous studies, the high-5HT animals had a significantly higher platelet 5HT level (Figure 2A) and body weight (Figure 2B) than the low-5HT animals. The BAT characteristics of animals from 5HT sublines in basal conditions, i.e., when animals were kept at an ambient temperature commonly used for experimental rodents, are shown on Figure 2C–G. In young (prepubertal) male animals, the mass of the iBAT/animal was significantly higher in high-5HT animals compared to low-5HT animals—L/H ratio ranged between 0.69 and 0.86 over several time points (Figure 2C), whereas this ratio had a trend for reversal in adult animals (L/H = 1.05 to 1.29, Figure 2C). Relative iBAT mass (expressed in grams per body mass; Figure 2D), showed no differences between the 5HT sublines in juvenile animals, while in adults it was significantly higher in the low-5HT animals than in the high-5HT animals (L/H = 1.28 to 1.40, Figure 2D). Essentially the same results were obtained in female animals (Figure S1).

**Figure 3:**
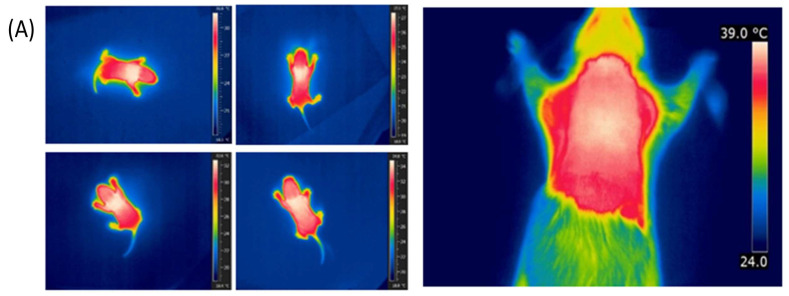

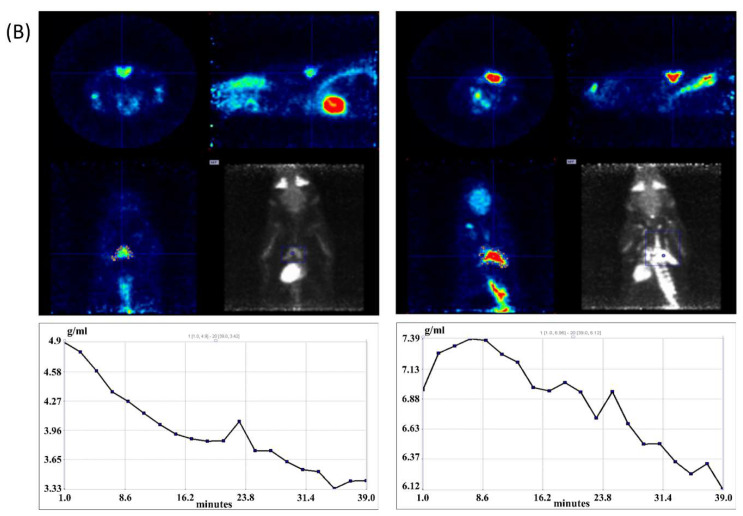
(**A**) Representative thermographic images of animals from the 5HT sublines. (**Left**): awake 7-day-old animals (upper: high-5HT, lower: low-5HT; there were no differences between the 5HT sublines; (**Right**): anesthetized 3.5-month-old animal from the low-5HT subline, differences between the 5HT sublines are shown in Section 3.3. (**B**) Representative image and plot of standard uptake values (SUV) in high-5HT animal (**left**) and low-5HT animal (**right**) obtained by FDG-micro PET imaging.

To compare the thermogenic activity of BAT in 5HT sublines, we measured the temperature over iBAT region by infrared thermography (Figure 2E), and determined the iBAT glucose uptake by FDG-microPET imaging (Figure 2F). In 3-month-old animals, both parameters were significantly higher in the low-5HT animals (Figure 2E,F), whereas in older (5 months) animals, only the iBAT temperature was higher in the low 5HT subline. The concentration of UCP1 protein, a BAT-specific thermogenic molecule, did not differ significantly between high-5HT and low-5HT animals at any of the ages studied, although in adults its levels were somewhat higher in the BAT of low-5HT animals (L/H = 1.19 to 1.39, Figure 2G). Representative thermographic and PET images are shown in Figure 3.

**Figure 4:**
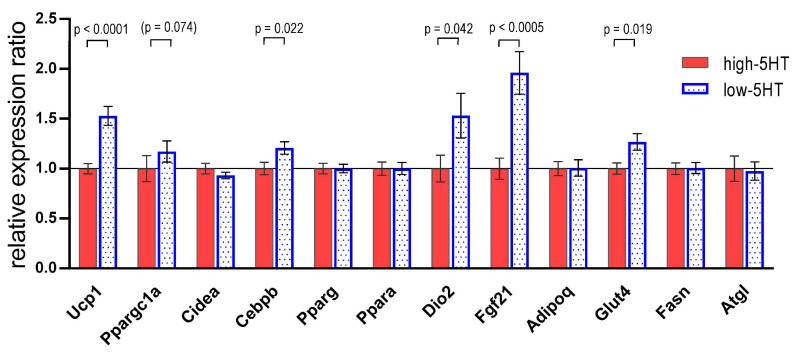
Expression of thermogenesis-regulating genes in interscapular brown adipose tissue of animals from 5HT sublines at 3–3.5 months of age, housed at an ambient temperature of 22 °C, measured by RT-qPCR analysis. Results are presented as the relative expression ratio between low-5HT and high-5HT subline (L/H), with high-5HT animals set at 1.0. Shown are means ± SEM, *n* = 9 (*Ppargc1a*, *Cidea*, *Adipoq*, *Atgl*) or 16/subline (other). The *p*-values were obtained by a *t*-test or MW test, as appropriate. An explanation of the abbreviations can be found at the end of the manuscript.

To investigate which molecules/pathways underlie the observed basal differences between 5HT sublines, we compared their iBAT expression levels of various genes involved in the regulation of thermogenesis and adipogenesis. We focused on BAT specific molecules/markers, transcription factors, and several signaling molecules, such as adipokines and growth factors, known to be associated with BAT function. As shown in Figure 4, the iBAT mRNA expression of several investigated genes, belonging to different classes of regulatory molecules, was upregulated in low-5HT animals compared to high-5HT animals. They are as follows: *UCP1* (L/H = 1.53), *Cebpb* (L/H = 1.21), *Dio2* (L/H = 1.53), *Fgf21* (L/H = 1.96), and *Glut4* (H/L = 1.21). There was a trend toward the upregulation of *Ppargc1a* (L/H = 1.17), whereas no differences were observed in the mRNA expression of *Cidea*, *Pparg*, *Ppara*, *Adipoq*, *Fasn*, and *Atgl*.

### 3.2. iBAT Functioning of 5HT Sublines in Conditions of Experimentally Induced Thermogenesis

To further assess potential differences in the iBAT functioning between animals from 5HT sublines, we conducted several functional measurements in conditions of experimentally induced thermogenesis.

**Figure 5:**
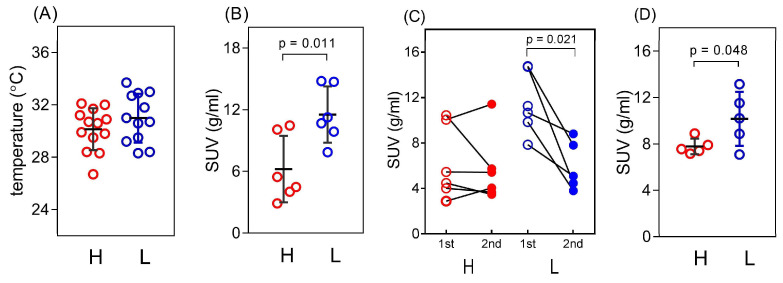
Characteristics of interscapular brown adipose tissue (iBAT) in animals of the high-5HT (H, red circles) and low-5HT (L, blue circles) subline after induction of thermogenesis by temperature or pharmacological challenge. (**A**) Temperature of the skin over iBAT of 7-day-old pups after exposure to a cold environment for one hour, measured with a thermal imaging infrared camera; *n* = 12–13/subline (**B**,**C**) iBAT glucose uptake of 5-month-old male animals after first (**B**) and first (open circles) and second (solid circles) (**C**) exposure to a cold environment for 24 h, as determined by ^18^F-fludeoxyglucose positron emission tomography (FDG-PET) imaging; (**D**) iBAT glucose uptake in 3-month-old males determined 30 min after i.p. injection of BRL 37344 at a dose of 1 mg/kg. *n* = 5–6/subline. Individual values and means ± SD are shown; *p*-values obtained by *t*-test are indicated. SUV = standard uptake value.

We first measured the temperature over the iBAT region in 7-day-old pups exposed for 1 h to low ambient temperature (17 °C). Consistent with findings in basal conditions (Figure 2E), no statistically significant differences were observed between male (Figure 5A) or female (not shown) pups from high-5HT and low-5HT subline, supporting the absence of important differences in the neonatal period.

Next, we measured iBAT glucose uptake, indicative of iBAT metabolic activity, in adult (5-month old) animals exposed for 24 h to low ambient temperature (12 °C). Consistent with findings in basal conditions (Figure 2F), levels of iBAT glucose uptake were significantly higher (by 1.86-fold) in low-5HT than high-5HT animals after the first episode of cold exposure (Figure 5B). However, no differences between the sublines were observed following the second episode of cold exposure 7 days after the first (Figure 5C), indicating differential adaptation of 5HT sublines to repeated cold exposure. Thus, it appears that in the low-5HT animals first cold exposure elicits a much stronger response than repeated exposure (*p* = 0.0209, paired *t*-test), while in high-5HT animals there is no difference between the first and second exposure.

Finally, we measured iBAT glucose uptake in 3-month old animals shortly (30 min) after the injection of a β-adrenergic agonist BRL 37344. Similarly to findings in basal conditions and after the 1st cold exposure, iBAT glucose uptake was significantly higher in low-5HT than high-5HT animals, confirming stronger iBAT functioning in this subline (Figure 5D). Taken together, these and basal findings clearly demonstrated differences in BAT functioning between animals with constitutively different serotonin tone.

### 3.3. Functional and Molecular Response to Chronic Cold Exposure

To better understand the functioning and regulation of BAT in 5HT sublines, we investigated functional and molecular BAT parameters in animals kept continuously at room temperature (H-RT, L-RT) or exposed to cold (11 °C) at regular intervals (H-CE, L-CE) for 5 weeks.

**Figure 6:**
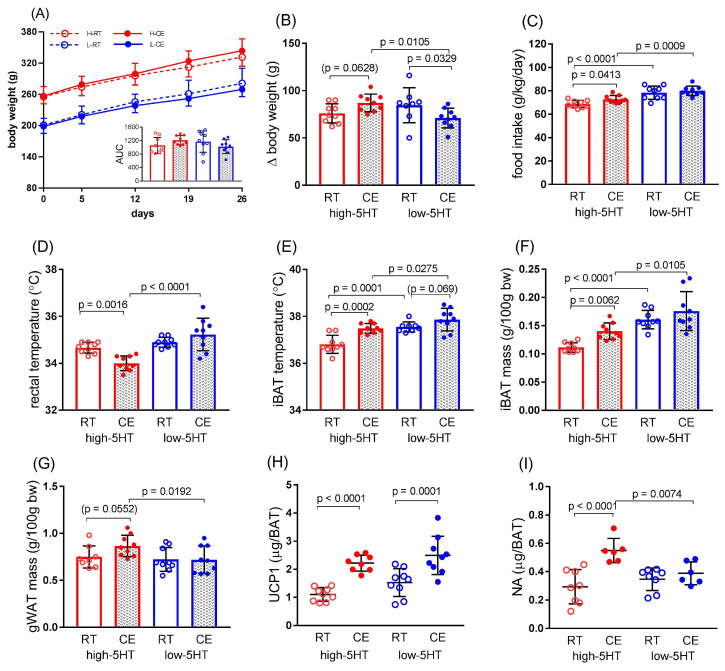
Functional response of high-5HT (red circles) and low-5HT (blue circles) sublines to repeated cold exposure. Animals at 2.5 months of age were kept continuously at room temperature (RT, open circles) or exposed to cold at regular intervals (CE, solid circles) for 5 weeks. (**A**) Body weight accumulation and corresponding area under curve values (AUC, inset graph); (**B**) Body weight gain and (**C**) food intake of the 5HT sublines measured during the fourth week of the experiment; (**D**) Average core body temperature of animals from 5HT sublines measured immediately after cold exposure on three consecutive days during the third week of the experiment; (**E**) Temperature of skin over the interscapular brown adipose tissue (iBAT) of 5HT sublines measured by infrared thermography at week four; (**F**,**G**) Mass of interscapular BAT and gonadal white adipose tissue (gWAT) determined in 5HT sublines at the end of experiment; (**H**) iBAT level of uncoupling protein1 (UCP1) and (**I**) noradrenalin (NA) measured by ELISA. Individual values and means ± SD are shown, *n* = 9 per group (except 4.I where *n* = 6–8 per group); *p*-values obtained by LSD or Dunn’s post-hoc test after 1w-ANOVA or KW test, respectively, are indicated.

The body weight of animals in all groups gradually increased with the time (Figure 6A). However, the body weight gain in the cold-exposed animals at the end of the experiment was significantly lower only in the low-5HT subline (L-CE/L-RT = 0.88; H-CE/H-RT = 1.14, Figure 6B). Consistent with this, the increase in food intake in CE animals compared with the corresponding RT animals (Figure 6C) was significantly increased only in the high-5HT subline.

**Figure 7:**
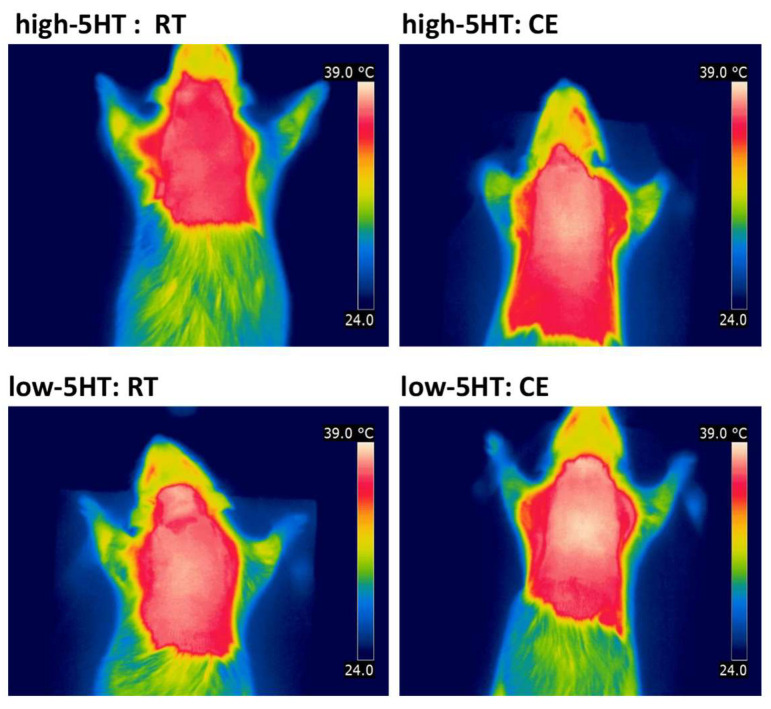
Representative thermographic images of the interscapular region of animals from the 5HT sublines obtained at room temperature (RT) or after cold exposure (CE), taken with a digital infrared camera.

Core body temperature, measured immediately after cold exposure, was also significantly lower only in the high-5HT subline (by 0.67 °C, Figure 6D). The iBAT temperature determined by infrared thermography was significantly increased by CE, compared with RT, in animals from the high-5HT subline (by 0.66 °C), whereas only a tendency to increase was observed in the low-5HT subline (by 0.32 °C; Figure 6E; representative thermographic images shown in Figure 7). Similarly, a significant increase in iBAT and a near significant increase in gWAT mass in response to CE was present only in high-5HT animals (iBAT: H-CE/H-RT = 1.26; gWAT: H-CE/H-RT = 1.16; Figure 6F,G). Cold exposure resulted in increased iBAT UCP1 content in animals from both 5HT sublines (H-CE/H-RT = 2.01, L-CE/L-RT = 1.63; Figure 6H), whereas iBAT noradrenaline was significantly different between the CE and RT groups only in the high-5HT subline (Figure 6I).

**Figure 8:**
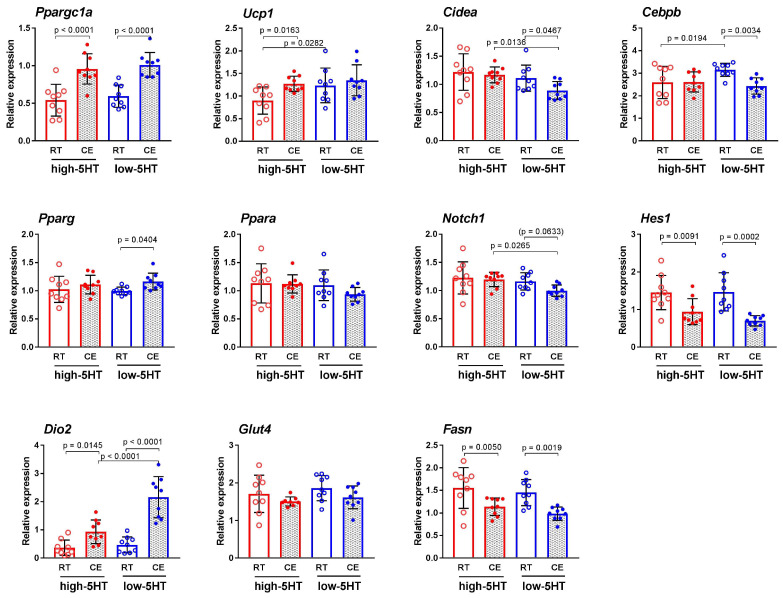
Expression of thermogenesis-related genes in the interscapular brown adipose tissue of animals from high-5HT (red circles) and low-5HT (blue circles) sublines at the age of ~3.5 months after constant maintenance at room temperature (RT, open circles) or intermittent exposure to cold environment (CE, solid circles) for 5 weeks, as obtained by RT-qPCR analysis. Individual values and means ± SD are shown; *n* = 9 rats per group; *p*-values obtained by LSD or Dunn’s post-hoc test after 1w-ANOVA or KW test, respectively, are indicated. An explanation of the abbreviations can be found at the end of the manuscript.

The expression levels of various classes of thermogenesis-related genes analyzed in iBAT after cold exposure of the animals are shown in Figure 8. CE exposure upregulated *Ppargc1a*, a master regulator of mitochondrial biogenesis, to a similar extent in both 5HT sublines (H-CE/H-RT = 1.77; L-CE/L-RT = 1.70). On the other hand, the increase in the mRNA level of thermogenic *Ucp1* was statistically significant only in animals from the high-5HT subline (H-CE/H-RT = 1.41; L-CE/L-RT = 1.10), whereas the mRNA level of *Cidea*, another BAT marker, was significantly decreased only in the low-5HT subline (L-CE/L-RT = 0.80).

Among genes encoding transcription factors, the expression of *Cebpb* was significantly decreased and the expression of *Pparg* was significantly increased only in the low-5HT animals compared with the corresponding RT group (Cebpb: L-CE/L-RT = 0.77, *Pparg*: L-CE/L-RT = 1.16), whereas the mRNA expression of *Ppara* did not differ between the CE and RT groups in either of the 5HT sublines. The expression of *Hes1* was significantly downregulated in both 5HT sublines, with more pronounced changes in the low-5HT animals (H-CE/H-RT = 0.65; L-CE/L-RT = 0.47). The expression level of *Fasn*, encoding a key enzyme of the lipogenesis pathways, was downregulated (H-CE/H-RT = 0.73; L-CE/L-RT = 0.67), and that of *Dio2*, encoding an enzyme in the thyroid pathway, was upregulated (H-CE/H-RT = 2.56, L-CE/L-RT = 4.61) in the CE groups of both 5HT sublines compared with corresponding RT groups. Again, the changes were more pronounced in low-5HT animals.

### 3.4. Functional and Molecular Response to Repeated Treatment with β_3_-Adrenergic Agonist

**Figure 9:**
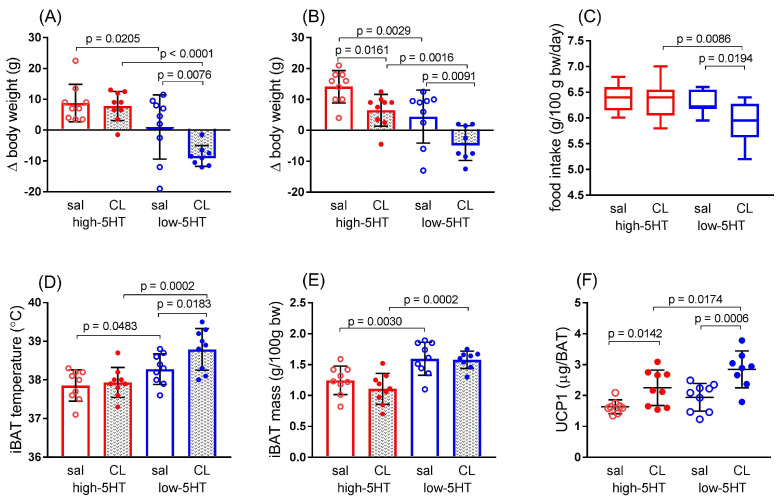
Functional response of high-5HT (red circles) and low-5HT (blue circles) sublines to pharmacological induction of thermogenesis. Animals from high-5HT and low-5HT sublines at the age of 3.5 months were treated daily with either CL316,243 (CL, solid circles) or saline (sal, open circles) for 6 consecutive days. (**A**,**B**) Changes in body weight after the (**A**) 4th and (**B**) 5th injections; (**C**) Food intake after the 5th injection; (**D**) Temperature of the skin over the interscapular brown adipose tissue (iBAT), measured by infrared thermography 2 h after the 5th injection; (**E**) iBAT mass determined after the 6th injection; (**F**) Uncoupling protein 1 (UCP1) level in iBAT at the end of the experiment. Individual values and means ± SD are shown, *n* = 8–9 per group; *p*-values obtained by LSD or Dunn’s post-hoc test after 1w-ANOVA or KW test are indicated.

We complemented functional and molecular studies on differences between 5HT sublines in cold exposure-induced thermogenesis by studies employing the pharmacological activation of thermogenesis with CL316,243 (CL), a specific β_3_-adrenergic agonist. Differences in body weight gain between the 5HT sublines became evident after only four days of treatment with CL (Figure 9A) and persisted after the 5th injection (Figure 9B). Thus, CL-treated animals of the low-5HT subline showed significantly lower weight gain compared to the saline-treated group and even showed a weight loss (Figure 9A,B). Accordingly, they also consumed less food (Figure 9C) and had an increased (by 0.51 °C) temperature over the iBAT region as determined by infrared thermography (Figure 9D). The BAT mass was not altered by the 6-day CL treatment in either of the sublines (Figure 9E), whereas the UCP1 protein level was increased by the CL treatment in animals of both 5HT sublines (H-CL/H-sal = 1.37, L-CL/L-sal = 1.46, Figure 9F).

**Figure 10:**
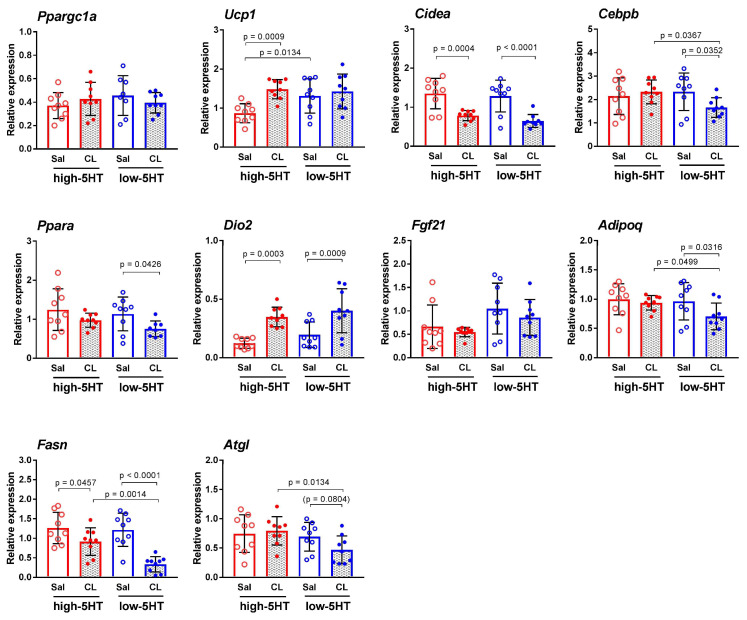
Expression of thermogenesis-related genes in interscapular brown adipose tissue of animals from high-5HT (red circles) and low-5HT (blue circles) sublines at the age of 3.5 months after six injections of CL316,243 (CL, solid circles) or saline (sal, open circles), as determined by RT-qPCR. Individual values and means ± SD are shown; *n* = 9 rats per group; *p*-values were obtained by LSD or Dunn’s post-hoc test after 1w-ANOVA or KW test, respectively. An explanation of the abbreviations can be found at the end of the manuscript.

The thermogenesis-related genes studied in iBAT after β_3_-adrenergic stimulation were partially the same as in the cold exposure experiment, with few additional genes analyzed. The changes of mRNA expression after pharmacological stimulation (Figure 10) were similar as after cold stimulation of thermogenesis. Thus, *Ucp1* expression in iBAT was significantly increased in response to CL treatment only in the high-5HT subline (H-CL/H-sal = 1.70), while expression of *Dio2* was upregulated (H-CL/H-sal = 2.82; L-CL/L-sal = 2.05) and expression of *Cidea* (H-CL/H-sal = 0.58; L-CL/L-sal = 0.50) and *Fasn* (H-CL/H-sal = 0.72; L-CL/L-sal = 0.27) was downregulated by CL treatment in both 5HT sublines.

Most of other genes studied were downregulated by CL316,243 treatment, significantly or by clear trend, only in the animals of the low-5HT subline. These were the following genes: *Cebpb* (L-CL/L-sal = 0.71), *Ppara* (L-CL/L-sal = 0.66), *Adipoq* (L-CL/L-sal = 0.73), *Glut4* (L-CL/L-sal = 0.43), and *Atgl* (L-CL/L-sal = 0.67). There were no differences in *Ppargc1a* and *Fgf21* mRNA expression between the CL-treated and saline-treated animals of either 5HT-subline.

## 4. Discussion

Our previous studies on the Wistar–Zagreb 5HT rat model have shown that constitutively elevated whole-body 5HT tone is associated with increased adiposity and impaired glucose and lipid metabolism [43,44,46]. They have also suggested that BAT thermogenesis may contribute to the observed metabolic differences between 5HT sublines [44,45]. The present study aimed to better characterize BAT biology of the 5HT sublines. We compared the 5HT sublines with respect to BAT thermogenic capacity/activity under baseline husbandry conditions and in response to physiological (cold exposure) and pharmacological (β_3_-adrenergic agonist) stimulations. A better understanding of how constitutive differences in the whole-body 5HT tone influence response to thermogenic challenges may be useful if the modulation of adaptive thermogenesis is to be used for therapeutic purposes, as proposed [2,4,5,49].

Baseline conditions in our studies refer to an ambient temperature of approximately 22 °C, which is standard in housing laboratory rodents, but is below the rat’s thermoneutral zone [50]. Living under these mild cold conditions results in the modulation of animal physiology, including the activation of BAT thermogenic mechanisms [51,52]. In adulthood, our animals from the high-5HT subline have a lower iBAT mass than the low-5HT animals. They also have lower iBAT metabolic activity, as evidenced by the lower iBAT temperature and iBAT glucose uptake. Juveniles of both 5HT sublines have a higher iBAT mass relative to body mass than adults, and this gradually decreases with age, as expected. At the juvenile age, no significant differences in relative iBAT mass and temperature were observed between the 5HT sublines, possibly suggesting that 5HT regulation of thermogenic mechanisms may not be fully established until the animal matures.

Our findings that BAT mass and function are higher in low-5HT animals than in high-5HT animals are consistent with pharmacological studies showing that the inhibition of peripheral 5HT synthesis increases BAT mass and thermogenesis [32,33]. They are consistent also with studies showing that drugs which increase 5HT bioavailability/activity both centrally and peripherally reduce BAT thermogenic activity [30,53]. This pharmacologically induced increase in whole-body 5HT activity is directly comparable to the situation in our high-5HT subline. It is therefore likely that both central and peripheral 5HT mechanisms jointly contribute to the observed differences in BAT functionality between 5HT sublines. Clinical studies generally associate decreased mass/activity of BAT with obesity [8,10,11,12], and these two features also co-occur in our high-5HT animals. However, the assumption that BAT is dysfunctional in obesity has recently been questioned [54,55].

The increased BAT thermogenic capacity/activity in low-5HT animals was associated with higher mRNA transcripts for some of the major markers/regulators of BAT thermogenesis, notably *Ucp1*, *Ppargc1a*, *Cebpb*, *Fgf21*, and *Dio2*. This suggests a role of 5HT signaling in regulating these genes in conditions of lifelong living in a mild cold environment. *Fgf21* mRNA expression in BAT was almost twice as high in the low-5HT animals as in the high-5HT animals. It appears therefore that Fgf21 is the most strongly regulated by constitutive 5HT tone of all thermogenic genes examined. Fgf21, recently recognized as an important metabolic regulator [56], is released from BAT upon cold stimuli to regulate Ppargc1a signaling and increase UCP1 expression [57,58,59]. The present results associate its regulation to a constitutive 5HT tone of the animal under mild thermogenic conditions.

It is known that BAT is involved in the regulation of systemic glucose and lipid homeostasis [13,35]. Our previous studies have shown functional differences in glucose and lipid metabolism between 5HT sublines and revealed some of the underlying central and peripheral mechanisms [43,44,46]. The present results, showing increased BAT mRNA expression of *Glut4* in low-5HT animals and no differences in *Fasn* and *Atgl* mRNA levels between 5HT sublines, suggest that 5HT regulation of BAT glucose, rather than lipid metabolism, may contribute to better metabolic health in animals from the low-5HT subline.

The differences in thermogenesis between 5HT sublines may be related to various peripheral or/and central 5HT mechanisms. For example, the lower BAT function observed in high-5HT animals could be due to a direct effect of 5HT on mitochondrial biogenesis. Specifically, it has been shown that 5HT inhibits the differentiation of preadipocytes into brown adipocytes and decreases the expression of key thermogenic markers in differentiated brown adipocytes [31]. Studies on our 5HT sublines are currently underway to determine 5HT levels and the expression of 5HT-regulating genes (enzymes, transporters, receptors) in cells from various adipose tissue depots, including iBAT. Preliminary results show that 5HT levels in iBAT are almost twice as high in high-5HT rats as in low-5HT rats (not published), so a direct inhibitory effect of 5HT on BAT functionality might be expected in high-5HT animals.

The differences in BAT function between 5HT sublines may also be mediated centrally, through 5HT modulation of the adrenergic system or some other mechanisms [35]. Experimentally induced increase in brain 5HT level has been shown to inhibit BAT sympathetic nerve activity and, in turn, BAT thermogenesis [5,60]. Further, increasing 5HT availability by the long term inhibition of the 5HT transporter decreased the spontaneous firing of noradrenergic neurons [61] and reduced thyroid signaling [62], which is required for an optimal BAT response to noradrenaline [63]. Our high-5HT rats are supposed to have increased brain 5HT activity compared to low-5HT animals (see Introduction), so a greater inhibition of BAT sympathetic activity in this 5HT subline is possible.

The differences in BAT thermogenesis between 5HT sublines might also be related to their developmental history. For example, early overfeeding is associated with BAT thermogenic hypoactivity and the downregulation of UCP1 in adulthood [64]. We have shown previously that pups from the high-5HT subline suckle more milk (15%) than their low-5HT counterparts [46].

Clearly, we cannot distinguish between the central and peripheral 5HT mechanisms responsible for thermogenic differences between 5HT sublines. However, their constitutively altered whole-body 5HT activity offers the advantage that various roles of 5HT signaling can be studied from an integral perspective, which is considered particularly important in studies of thermogenesis [65]. The twofold difference in platelet 5HTT activity between the 5HT sublines is similar to the degree of variation in 5HTT expression levels in the human population [66,67], so our model may well mimic the natural human situation. In spite of the numerous important differences between rodents and humans, their brown adipocytes share similar cell physiology and thermogenic activity [68]. Therefore, our WZ-5HT sublines may be a useful model to study the role of 5HT signaling in the (dys)regulation of thermogenesis.

As discussed above, animals from 5HT sublines living in a mild cold environment developed a different adaptive thermogenesis. Further experiments with stronger cold stimuli or specific pharmacological challenges revealed additional functional and molecular differences between sublines.

The cold exposure increased food intake, as a compensatory response to the increased heat demand [69,70], only in the high-5HT animals. Previously, we have shown that the high-5HT animals have significantly higher daily food intake when the absolute amount of consumed food is considered, while their daily food intake adjusted for body weight is significantly lower than in low-5HT rats [46]. The high-5HT animals exposed to cold also exhibited a higher body weight gain compared to the corresponding controls, which may accord with the view that while cold exposure can improve metabolic health, this does not necessarily lead to weight loss [49]. In contrast, the low-5HT animals showed no increase in food intake and had significantly lower weight gain compared to the corresponding controls, both indicating a better functional thermogenic response.

Unlike the high-5HT animals, the low-5HT animals exposed to cold did not show a decrease in core body temperature at the time point measured (third week of the experiment). They also underwent shivering thermogenesis (visually monitored by the experimenter) which likely contributed to their ability to better control core body temperature in cold environment. Using a protocol similar to ours, Wang et al. [71] have shown that intermittent cold exposure provokes an early drop in rectal temperature, which gradually decreases with repeated daily exposure and finally disappears after approximately 14 days. It is possible that the respective cold adaptation develops more slowly in high-5HT than in low-5HT animals. It is well known that 5HT neurons are critically involved in thermoregulation [35,72]. Thus, the hypothermic response observed in high-5HT animals may be centrally mediated and related to their higher brain 5HT activity.

The CL316,243 experiment essentially replicated the results of the cold exposure experiment. When all experiments with various thermogenic challenges are considered together, it is clear that the metabolic/functional activity of BAT is higher in low-5HT animals than in high-5HT animals. In particular, iBAT glucose uptake was higher in low-5HT than in high-5HT rats after both 24-h cold exposure and acute BRL 37344 treatment, and the iBAT temperature was higher in low-5HT than in high-5HT rats after both 4 weeks of repeated cold exposure and 5 days of CL316,243 treatment.

In summary, all functional indices examined (i.e., food intake, body weight change, core body temperature, shivering thermogenesis, iBAT temperature, and iBAT glucose uptake) suggest, with more or less certainty, that low-5HT animals have a better functional response to various thermogenic challenges than high-5HT animals. Although this trait may be functionally related to the leanness of low-5HT animals in comparison to the fatness of their counterparts, both phenotypes, i.e., thermogenic capacity and leanness/fatness, are related to endogenous 5HT activity. We hypothesize that increased constitutive 5HT activity is associated with a poorer ability of the organism to cope with thermogenic challenges. The results may also indicate that individuals with lower 5HT activity may benefit more from treatment with β-adrenergic agents.

Gene expression data on molecules known to be physiological regulators of thermogenesis shed some light on the mechanisms responsible for the observed differences between the 5HT sublines. Thus, both repeated cold exposure and CL316,243 treatment increased iBAT protein levels of UCP1 (a key marker of adaptive thermogenesis) in both 5HT sublines, while *UCP1* mRNA levels were significantly upregulated by the respective challenges only in the high-5HT subline (Figure S2). Notably, at a standard housing temperature (22 °C), *UCP1* mRNA levels were significantly higher in low-5HT than in high-5HT rats, suggesting that the regulation of *UCP1* expression by 5HT may depend on the duration and intensity of the cold stimulus (lifelong mild vs. intermittent stronger). The lack of differences in *Ucp1* mRNA levels between the H-CE and L-CE groups and between the H-CL and L-CL groups can probably be explained by the pre-existing elevated *Ucp1* mRNA levels in the low-5HT animals. However, it should be noted that the corresponding Ucp1 protein levels were higher in the L-CE and L-CL groups than in the H-CE and H-CL groups, respectively, which is consistent with the better thermogenic function of the low-5HT animals.

Furthermore, in both sublines, cold exposure upregulated the mRNA expression of *Ppargc1a* (transcriptional coactivator of mitochondrial biogenesis) and *Dio2* (thyroid hormone-activating enzyme) and downregulated the mRNA expression of *Fasn* (fatty acid-synthesizing enzyme) and *Hes1* (transcription factor in the Notch pathway). However, mRNA expression of most other genes studied was markedly, positively (*Pparg*) or negatively (*Cidea*, *Cebpb*, *Ppara*, *Notch1*, *Adiponectin*), regulated by cold exposure only in the low-5HT animals, further supporting the better BAT thermogenic functions in animals from the low-5HT subline compared to high-5HT subline. Cold-induced changes in mRNA levels of key thermogenic genes are reported also in other cold exposure studies [52,57,73,74,75], while a few genes were investigated here for the first time in this context (*Notch1*, *Hes1*). In contrast to repeated cold exposure, which induced upregulation of *Ppargc1a* mRNA in both 5-HT sublines, treatment with CL316,243 did not induce changes in *Ppargc1a* mRNA levels in either subline. This suggests differences in the regulation of *Ppargc1a* in the context of the different thermogenic challenges in our animals, and the same seems to be true for *Cidea*, another BAT marker.

Extreme physiological heterogeneity in BAT capacity and function has been noted in humans, emphasizing the need to identify the causes of this variability [49,76]. Several factors have been shown to strongly influence BAT activity/regulation, such as the circadian rhythm [77] or the timing of feeding/fasting [78], and our results demonstrated that thermogenesis is also modulated by constitutive 5HT activity, at least in rats.

Some of the thermogenesis-related genes which were upregulated in low-5HT animals compared to high-5HT rats under basal (lifelong mild cold) conditions, such as *Glut4* and *Fgf21*, and which would be expected to increase expression after induction of thermogenesis by more severe cold or pharmacological stimulation, did not actually show changes in the mRNA levels in cold exposure or CL316,243 experiments. With regard to *Glut4*, the data in the literature are rather ambiguous. Thus, in addition to reports showing a marked increase in its expression in parallel with the increase in cold-induced glucose uptake [79,80], there are also studies showing that the increase in Glut4 expression depends on the duration of the cold exposure [81] or that there are no changes in Glut4 expression after thermogenesis induction [82]. In addition, a marked decrease in *Glut4* mRNA levels was observed in mature brown adipocytes treated in vitro with CL316,243 [83]. Further, in a study by [84], systemic administration of beta-adrenergic agents significantly inhibited cold-induced overexpression of Glut4 in BAT, adding to the complexity of glucose regulating mechanisms in BAT.

It should be noted that we analyzed gene expression only at the mRNA level. Although concordant changes in mRNA and protein levels have been reported for some of the genes of interest [31,57], this limitation should be taken into account when interpreting our results.

It should be noted that the observed differences between the 5HT sublines in BAT functioning under different thermogenic challenges are probably influenced also by their differential coping with the mild cold environment in which they live. The number of studies emphasizing that housing temperature should be considered when interpreting thermogenic results has recently increased [51,76,85].

## 5. Conclusions

In conclusion, our results obtained in the genetic model of rats with constitutively different whole-body 5HT tone, confirm some known and suggest some new interrelations between 5HT activity and the thermogenic responsiveness of the BAT. In particular, we have shown that animals with constitutively low 5HT activity maintained at standard housing temperature (22 °C) have a greater iBAT mass and higher iBAT metabolic activity (iBAT temperature and glucose uptake), accompanied by the increased iBAT mRNA expression of key thermogenic genes. In response to further thermogenic challenges—intermittent cold exposure or treatment with a β_3_-adrenergic agonist—5HT sublines show a number of functional and molecular differences linking constitutively low endogenous 5HT tone to higher BAT activity/capacity. They also suggest that individuals with lower 5HT activity may be more sensitive to β_3_-adrenergic drugs, which may be important from a clinical perspective.

Considering that our 5HT sublines with constitutive differences in 5HT tone may well mimic the physiological situation in humans and that rodent and human brown adipocytes share similar cell physiology and thermogenic activity [68], WZ-5HT rats could likely provide a valuable translational model to study the role of 5HT mechanisms in the (dys)regulation of thermogenesis from the integral perspective.

## Data Availability

All data generated for this study are included in the article.

## Supplementary Materials

The following supporting information can be downloaded at: https://www.mdpi.com/article/10.3390/life13071436/s1, Table S1. Primer sequences used in RT-qPCR analysis; Figure S1. Mass of interscapular brown adipose tissue in female animals from high-5HT and low-5HT sublines; Figure S2. UCP1protein and mRNA levels in BAT of high-5HT and low-5HT animals after cold exposure and CL316,214 treatment.

## Abbreviations

*5HTT*, serotonin transporter; *Actb*, actin beta; *Adipoq*, adiponectin; *Atgl*, adipose triglyceride lipase; *CE*, cold exposure; *Cebpb*, CCAAT/enhancer binding protein beta; *Cidea*, cell death inducing DFFA like effector A; *Dio2*, iodothyronine deiodinase 2; *Fasn*, fatty acid synthase; *FDG-PET*, [^18^F]fludeoxyglucose positron emission tomography; *Fgf21*, fibroblast growth factor 21; *Glut4*, glucose transporter4; *gWAT*, gonadal white adipose tissue; *H*, high-5HT subline; *Hes1*, Hes family bHLH transcription factor 1; *iBAT*, interscapular brown adipose tissue; *L*, low-5HT subline; *NA*, noradrenalin; *Notch1*, neurogenic locus notch homolog1; *Ppara(g)*, peroxisome proliferator-activated receptor alfa (gamma); *Ppargc1a*, pparg-gamma coactivator 1 alpha; *PSL*, platelet serotonin level; *PSU*, platelet serotonin uptake; *RT*, room temperature; *SUV*, standard uptake values; *Tph1*, tryptophan hydroxylase1; *Ucp1*, uncoupling protein1.
