# Supplementary material for: Constitutive Serotonin Tone as a Modulator of Brown Adipose Tissue Thermogenesis: A Rat Study
Source: Life (Basel). 2023 Jun 25;13(7):1436. doi: 10.3390/life13071436 (PMC10381595; doi:10.3390/life13071436)
Supplement: Supplementary file 1 [file life-13-01436-s001.zip › life-2408081-supplementary.pdf]

Supplementary material

Table S1. Primer sequences used in RT-qPCR analysis.

NCBI Symbol	Gene	Forward primer sequence	Reverse primer sequence
**Actb**	Actin beta	GCGCAAGTACTCTGTGTGGA	GGCATCATTGACGACATCGAG
**Adipoq**	Adiponectin	GAGACGCAGGTGTTCTTG	CCTACGCTGAATGCTGAG
**Atgl**	Adipose triglyceride lipase	AGACTGTCTGAGCAGGTGGA	AGTAGCTGACGCTGGCATTC
**Cebpb**	CCAAT enhancer binding protein beta	GACAAGCTGAGCGACGAGTA	AGCTGCTCCACCTTCTTCTG
**Cidea**	Cell Death Inducing DFFA Like Effector A	TGACATTCATGGGGTTGCAGA	GGCCAGTTGTGATGACCAAGA
**Dio2**	Iodothyronine deiodinase 2	AGAAGTGGGAGTTGGCTTCG	ACTCTCTGACCGGATGACGA
**Fasn**	Fatty acid synthase	GGTAGGCTTGGTGAACTGTCTC	TCTAACTGGAAGTGACGGAAGG
**Fgf21**	Fibroblast growth factor 21	AGGCTTTGACACCCAGGATT	ACAGATGACGACCAGGACAC
**Glut4**	Glucose transporter 4	ATCAACGCCCCACAGAAAGT	CCTGCCTACCCAGCCAAGT
**Hes1**	Hes family bHLH transcription factor 1	CGACACCGGACAAACCAAA	GAATGTCTGCCTTCTCCAGCTT
**Notch1**	Neurogenic locus notch homolog	ATGACCTAGGCAAGTCAGCTC	ATTCATCCAAAAGCCGCACG
**Pparα**	Peroxisome proliferator activated receptor alpha	CCCTCGGAGAGGAGAGTTCC	GCTGGAGAGAGGGTGTCTGT
**Pparg**	Peroxisome proliferator activated receptor gamma	TCGCTGATGCACTGCCTATG	TGATTCCGAAGTTGGTGGGC
**Ppargc1a**	PPARG coactivator 1 alfa	GGACATGTGCAGCCAAGACTCT	CACTTCAATCCACCCAGAAAGCT
**Ucp1**	Uncoupling protein 1	GGGCTGATTCCTTTTGGTCTCT	GGGTTGCACTTCGGAAGTTGT

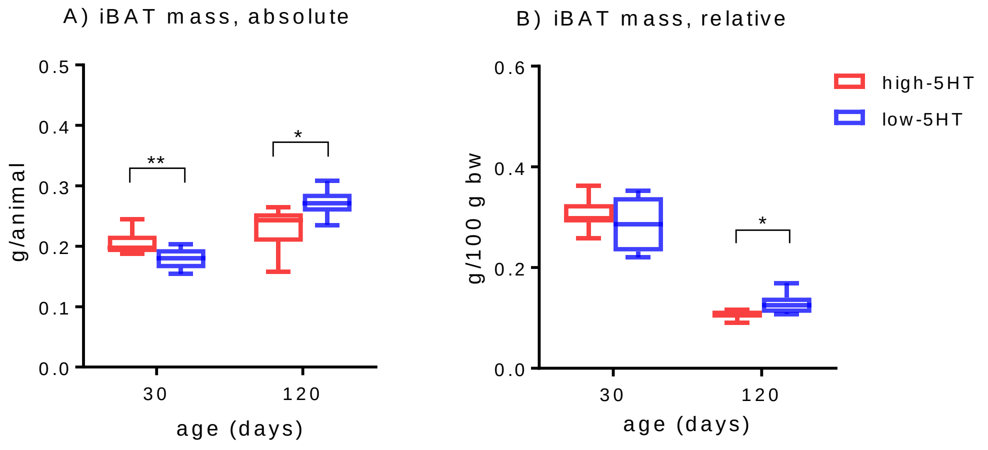

Figure S1. Mass of interscapular brown adipose tissue (iBAT) in female animals from high-5HT and low-5HT sublines housed at an ambient temperature of 22°C, expressed as weight per animal (A) or weight per body mass (B). Data are presented as median, interquartile range and min to max; n = 6-9/group; *p<0.05, **p<0.01, p-values obtained by t-test.

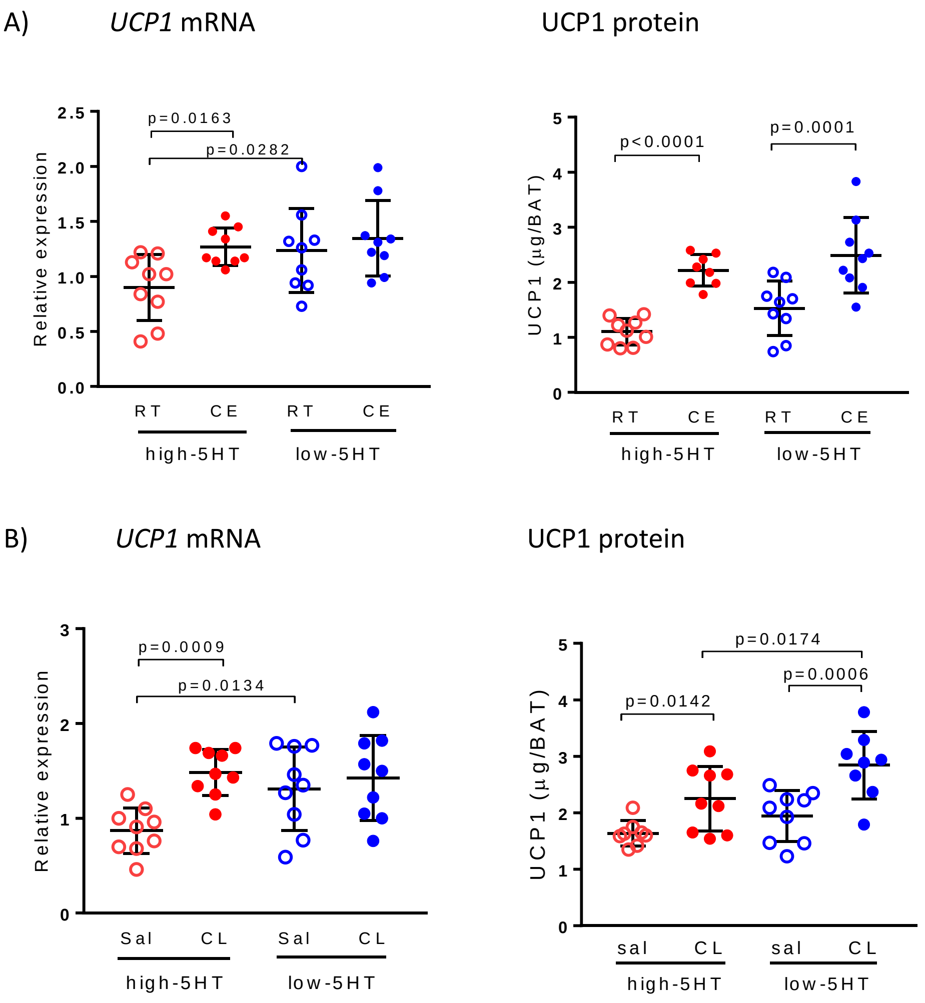

Figure S2. UCP1 protein and mRNA levels in BAT of high-5HT and low-5HT animals after **A**) cold exposure and **B**) CL316,214 treatment. Same data as shown in Figures 6 and 8 (cold exposure) and Figures 9 and 10 (CL treatment).
